# Differential Acclimation of Enzymatic Antioxidant Metabolism and Photosystem II Photochemistry in Tall Fescue under Drought and Heat and the Combined Stresses

**DOI:** 10.3389/fpls.2016.00453

**Published:** 2016-04-14

**Authors:** Aoyue Bi, Jibiao Fan, Zhengrong Hu, Guangyang Wang, Erick Amombo, Jinmin Fu, Tao Hu

**Affiliations:** ^1^Wuhan Botanical Garden, Chinese Academy of SciencesWuhan, China; ^2^University of Chinese Academy of SciencesBeijing, China; ^3^China-Africa Center, Chinese Academy of SciencesBeijing, China

**Keywords:** tall fescue, drought stress, heat stress, antioxidant enzymes, chlorophyll a fluorescence (OJIP), gene expression

## Abstract

Quality inferiority in cool-season turfgrass due to drought, heat, and a combination of both stresses is predicted to be more prevalent in the future. Understanding the various response to heat and drought stress will assist in the selection and breeding of tolerant grass varieties. The objective of this study was to investigate the behavior of antioxidant metabolism and photosystem II (PSII) photochemistry in two tall fescue genotypes (PI 234881 and PI 578718) with various thermotolerance capacities. Wide variations were found between heat-tolerant PI 578718 and heat-sensitive PI 234881 for leaf relative water content, malondialdehyde and electrolyte leakage under drought, high-temperature or a combination of both stresses. The sensitivity of PI 234881 exposed to combined stresses was associated with lower superoxide dismutase activity and higher H_2_O_2_ accumulation than that in PI 578718. Various antioxidant enzymes displayed positive correlation with chlorophyll content, but negative with membrane injury index at most of the stages in both tall fescue genotypes. The JIP-test analysis in PI 578718 indicated a significant improvement in ABS/RC, TR_0_/RC, RE_0_/RC, RE_0_/ABS values as compared to the control regime, which indicated that PI 578718 had a high potential to protect the PSII system under drought and high temperature stress. And the PS II photochemistry in PI 234881 was damaged significantly compared with PI578718. Moreover, quantitative RT-PCR revealed that heat and drought stresses deduced the gene expression of *psbB* and *psbC*, but induced the expression of *psbA*. These findings to some extent confirmed that the various adaptations of physiological traits may contribute to breeding in cold-season turfgrass in response to drought, high-temperature, and a combination of both stresses.

## Introduction

Genetically diverse plant populations display variation response patterns under various abiotic environments (Baxter and Dilkes, 2012). Therefore, a better understanding of differential adaptations to the diverse environment among different plant species with different abiotic tolerant can provide crucial information to improve the crop breeding process. Drought, high temperature and a combination of both stresses are the major abiotic stresses which limit the plant growth (Huang and Gao, 1999), which gives rise to a series of morphological, physiological, biochemical, and molecular changes that adversely influence plant growth and productivity (Wang et al., 2001).

Drought, high-temperature, and a combination of both stresses can cause the changes in turf quality as well as leaf water potential, cell membrane stability, photosynthetic rate, photochemical efficiency, and carbohydrate accumulation (Huang et al., 1998; Huang and Gao, 1999; Jiang and Huang, 2000). It was reported that drought and heat stresses limited CO_2_ uptake in bean (*Phaseolus vulgaris* L.) (Yordanov et al., 1997), leaf growth in sorghum [*Sorghum bicolor* (L.) Moench] (Kaigama, 1982), and leaf water content and potential in wheat (*Triticum aestivum* L.) (Shah, 1992). These diverse environmental stresses often result in activation of similar cell signaling pathways (Shinozaki and Yamaguchi-Shinozaki, 2000; Knight and Knight, 2001; Zhu, 2001, 2002), and cellular responses, such as the production of stress proteins, up-regulation of anti-oxidants, and accumulation of compatible solutes (Cushman and Bohnert, 2000).

Plants respond to the stress-induced production of reactive oxygen species (ROS) by changing component quantities of their defensive system (Zabalza et al., 2008). As mentioned, above, ROS consist of non-radical (H_2_O_2_) and free radical species (${\text{O}}_{2}^{-∙}$, OH^∙^, ${\text{OH}}_{2}^{∙}$). High ROS concentration is potentially detrimental to plants cells. ROS accumulation will have a significant impact to indispensable biomolecules [e.g., DNA, proteins, lipids, chlorophyll (Chl) and membrane] (Blokhina et al., 2003). Higher plants normally protect cells and subcellar systems against the cytotoxic effect by a complex antioxidant non-enzymatic and enzymatic systems (Ali et al., 2008). Enzymatic antioxidants consist of superoxide dismutase (SOD), catalase (CAT), and peroxidase (POD), which can remove H_2_O_2_, neutralize or scavenge free radicals and oxy-intermediates (Karpinski and Muhlenbock, 2007; Lee et al., 2007). Other studies have indicated that young senescing leaf cells excessively produced ROS under stressful conditions, which are eventually removed by complex non-enzymatic compounds (i.e., ascorbate, glutathione, α-tocopherol) and enzymes (i.e., CAT, SOD, glutathione POD, and glutathione reductase) (Scandalios et al., 1997). The denominator in all the adverse conditions is the imbalance between pro-oxidants and antioxidants disruption of homeostasis (Pinheiro et al., 2004). Apart from their detrimental effects on cells, ROS can simultaneously function as signaling molecules in reaction cascades activated by the external and developmental stimuli (Demiral et al., 2011). Therefore, it has been major focus area for further research. However, the specific antioxidant mechanisms involved in abiotic stress tolerance has not been fully elucidated.

Photosystem II (PSII), with its oxygen-evolving complex (OEC), is one of the three main stress-sensitive segments in the photosynthetic machinery (Mohanty et al., 2007; Murata et al., 2007). PSII is the core portion of the photosynthesis process which consists of a multi-subunit complex, and is embedded in the thylakoid membranes of higher plants. PSII catalyzes the dissociation of water into dioxygen and reducing equivalents by solar energy (Umena et al., 2011). The PSII reaction center is composed of a D1–D2 heterodimer, which binds all the essential cofactors for the electron transfer from the water-splitting complex to the plastoquinone (PQ) pool producing excess ROS (Pfannschmidt, 2003; Pospíšil, 2012). PSII is vulnerable to heat stress, which results in serious damage on PSII components (Hideg et al., 2002).

Tall fescue (*Festuca arundinacea* Schreber) is a wind-pollinated, self-infertile polyploid (2n = 6x = 42 chromosomes) perennial cool-season forage and turf grass. This botanical species is one of the most vital and intensively studied turf species globally (Wang et al., 2004). In the present study, two tall fescue accessions PI 234881 and PI 578718 with different high-temperature tolerance, whose thermotolerance was identified through the summer adaptation test in Wu Han for 2 years, were used to investigate response under high-temperature stress (Hu et al., 2015). The present work was conducted to investigate the variation acclimation of enzymatic antioxidant metabolism and PSII photochemistry in response to drought, heat, and the combined stress for tall fescue genotypes differing in high-temperature tolerance.

## Materials and methods

### Plant materials

This study was conducted at Wuhan Botanical Garden, Chinese Academy of Science, Wuhan, China in 2014. Two tall fescue accessions PI 234881 and PI 578718 were seeded in plastic pots (13 cm in diameter and 15 cm deep) with nutrient soil. Seedlings were grown for 7 days under controlled conditions (light/dark regime of 14/10 h at 22/18°C, relative humidity of 70%, photosynthetic photon flux density of (PAR) 360 μmol.m^−2^.s^−1^) and were sub-irrigated every other day with a half-strength Hoagland's solution (Hoagland and Arnon, 1950).

### Stress treatments

After 7-day of pre-adaptation, tall fescue with similar growth rate was arranged in a randomized complete block design with four replicates. The experiment included two temperatures and two soil moisture regimes. Temperature treatments were optimum (22°C/18°C, day/night) and high (35°C/30°C). Soil moisture treatments were (i) well-watered, i.e., irrigating every day until there was free drainage at bottom of the pots (ii) drought stress, i.e., withholding irrigation at optimum temperature. The treatments were defined as follows: (i) control: optimum soil moisture and temperature; (ii) drought: low soil moisture and optimum temperature (for 7-day stress until the soil moisture was lower than 30%); (iii) heat: optimum soil moisture and high temperature (for 6-day normal control and 1-day high temperature); (iv) drought + heat: low soil moisture and high temperature. Soil moisture was monitored with time domain reflectometry (TDR, Soil moisture Equipment Corp., CA; for 6-day drought stress and 1-day drought and high temperature stress). All groups were harvested on the 14th day. Fully expanded 3rd leaves of tall fescue were collected and stored at −80°C for subsequent analysis.

### Measurements

#### Chlorophyll a fluorescence transient

Fully expanded 3rd leaves (from bottom) were used for fluorescence measurements. All measurements were conducted by a pulse-amplitude modulation (PAM) fluorometer (PAM 2500, Heinz Walz GmbH) with high time resolution (10 μs). The leaves were stored under dark conditions for 30 min using leaf clips and then saturating light intensity was set to 2000 μmol photons m^−2^ s^−1^ (sufficient excitation intensity to insure closure of all PSII reaction centers to obtain a true fluorescence intensity of FM). The leaves were exposed to the strong light for 5 s (Korres et al., 2003). Finally fluorescence curves extending from minimal fluorescence (Fo) to maximal fluorescence (Fm) were produced by the OJIP transient. For each group, measurements were repeated at least four times. The PSII parameters and OJIP transient were analyzed according to Strasser et al. (2004).

#### The JIP-test

The fluorescence intensity emitted by plants changed over time. When the tall fescue was exposed to light from the dark adaption, fluorescence intensity increased and then declined. Chlorophyll fluorescence kinetics curve referred to the changing processfrom point O to point P and a typical JIP-test included four phrase: O-J (0.05~5 ms), J-I (5~50 ms), and I-P (50~1000 ms) which is based on the energy fluxes in biofilm. This provided a convenient tool for the further investigation of photosynthesis. The energy flow begins by the absorption (ABS) of light by PSII antenna pigments and ends with the reduction of the end electron acceptors (RE) at the Photosystem I (PSI) electron acceptor side driven by PSI (Stirbet, 2011). Detailed information of the introduced parameters is listed in Table 1.

**Table 1:** **Photosynthetic parameters deduced by the JIP-test analysis of fluorescence transients**.

	**C**		**D**		**H**		**H**+**D**		**Definitions**
	**PI234881**	**PI578718**	**PI234881**	**PI578718**	**PI234881**	**PI578718**	**PI234881**	**PI578718**	
**DATA EXTRACTED FROM THE RECORDED OJIP FLUORESCENCE TRANSIENT CURVES**									
*F*_0_ = *F*_20μ*s*_	0.54b	0.28a	0.68a	0.27a	0.57b	0.27a	0.67a	0.29a	Fluorescence at time t after onset of actinic illumination
*F*_K_	1.17b	0.63ab	1.32a	0.65a	1.2953a	0.60c	1.33a	0.61bc	Fluorescence value at 300 μs
*F*_J_	1.26b	0.74a	1.39a	0.75a	1.405a	0.68b	1.41a	0.69b	Fluorescence value at the J-step (2 ms) of OJIP
*F*_I_	1.73a	1a	1.69a	1.02a	1.79a	0.89b	1.68a	0.88b	Fluorescence value at the I-step (30 ms) of OJIP
*F*_*P*_ ≡ *F*_*M*_	2.16a	1.16a	2.06a	1.15a	2.07a	1.06b	2.02a	1.04b	Fluorescence value at the peak of OJIP test
*M*_0_	1.57b	1.59b	1.86a	1.72a	1.93a	1.65ab	1.91a	1.72a	Approximate value of the initial slope of fluorescence transient curves
**SPECIFIC ENERGY FLUXES (PER ACTIVE PSII REACTION CENTER)**									
ABS/RC	4.69b	4.01b	5.49a	4.08b	4.81b	4.25ab	5.48a	4.48a	Absorbed photon flux per RC
TR_0_/RC	3.50a	3.04b	3.63a	3.13ab	3.48a	3.17ab	3.57a	3.2a	Trapped excitation flux (leading to QA reduction) per RC
ET_0_/RC	1.94a	1.45a	1.77b	1.41a	1.54c	1.53a	1.66bc	1.48a	Electron transport flux (further than QA-) per RC
RE_0_/RC	0.93a	0.57b	1.04a	0.48c	0.65b	0.7a	0.93a	0.69a	Electron flux reducing end electron acceptors at the PSI acceptor side, per RC
**QUANTUM YIELDS AND EFFICIENCIES/PROBABILITIES**									
φ_P0_ ≡ TR_0_/ABS	0.75a	0.76a	0.66b	0.77a	0.72a	0.75ab	0.65b	0.72b	Maximum quantum yield for primary photochemistry, namely F_V_/F_M_
ψ_E0_ ≡ ET_0_/TR_0_	0.55a	0.48a	0.49b	0.45a	0.44c	0.48a	0.46bc	0.46a	Efficiency/probability with which a PSII trapped electron is transferred from Q_A_ to Q_B_
φ_E0_ ≡ ET_0_/ABS	0.41a	0.36a	0.33b	0.35ab	0.32b	0.36ab	0.3b	0.33b	Quantum yield of the electron transport flux from Q_A_ to Q_B_
δ_R0_ ≡ RE_0_/ET_0_	0.48b	0.39b	0.59a	0.34c	0.42b	0.46a	0.56a	0.47a	Efficiency/probability with which an electron from Q_B_is transferred until PSI acceptors
φ_R0_ ≡ RE_0_/ABS	0.2a	0.14b	0.19a	0.12c	0.136c	0.16ab	0.17b	0.17a	Quantum yield for reduction of end electron acceptors at the PSI acceptor side
γ_RC_	0.18a	0.2a	0.15b	0.19a	0.17a	0.19ab	0.15b	0.18b	Probability that a PSII Chl molecule functions as RC
RC/ABS	0.21a	0.25a	0.18b	0.25ab	0.20a	0.24ab	0.18b	0.23b	Number of Q_A_ reducing RCs per PSII antenna Chl
**PERFORMANCE INDEXES (PI, COMBINATION OF PARAMETERS)**									
PI_ABS_	0.79a	0.73a	0.36b	0.67ab	0.44b	0.66ab	0.30b	0.52b	PI (potential) for energy conservation from exciton to the reduction of intersystem electron
PI_total_	0.72a	0.48a	0.50b	0.35b	0.33c	0.56a	0.38c	0.44ab	PI (potential) for energy conservation from exciton to the reduction of PSI end acceptors

*The calculation of each parameter is based on the method described by Yusuf et al. (2010). Subscript “0” indicates that the parameter refers to the onset of illumination. Values are given as the average of 4 replicates, and different letters indicate statistical difference significance at P < 0.05 among the treatments by Duncan's multiple range tests*.

#### Leaf relative water content

After the stress treatments, fully expanded 3rd leaves of tall fescue were collected to determine the fresh weight (FW). The leaves were then stored in demonized water for 12 h under room temperature. Subsequently, turgid weights (TW) were determined, and then the leaves were put in oven at 80°C for 3 days. The dry weight (DW) was measured and followed by calculation of relative water content (RWC) using this formula:

$$\begin{array}{l} RW\left(\%\right)=\frac{FW-DW}{TW-DW}\times 100 \end{array}$$

#### Chlorophyll content

To determine the leaf Chl content, we employed the method described by Hiscox and Israelstam (1979). Fresh leaves (0.1 g) were sheared and soaked in a tube. A 10 mL dimethylsulfoxide was added and the contents were kept in darkness for 72 h. Finally, the absorbance of samples at 645 and 663 nm was measured with spectrophotometer (UV-2600, UNICO, Shanghai).

#### Lipid peroxidation

Lipid peroxidation was measured in terms of MDA content described by Heath and Packer (1968). One ml of supernatant was added in 4 ml of 20% (v/v) trichloroacetic acid containing 0.5% (v/v) thiobarbituric acid. The mixture was incubated at 95°C for 30 min in a water bath and cooled quickly in an ice bath. The aliquots were centrifuged at 10,000 × g for 10 min and absorbance of the supernatant was read at 532 nm. The value for the non-specific absorption at 600 nm was subtracted from the 532 nm reading. Blank contained complete reaction mixture without enzyme solution. The concentration of MDA was calculated by MDA's extinction coefficient of 155 mM^−1^ cm^−1^.

#### Electrolyte leakage

To determine electrolyte leakage (EL), about 0.1 g of random sampling leaves were washed with deionized water three times and cut into 0.5 cm long fragments. The test tubes were filled with 15 ml deionized water and shaken for 24 h at 25°C. The initial conductivity (Ci) was measured using a conductivity meter (JENCO-3173, Jenco Instruments, Inc., San Diego, CA, USA). The test tubes were autoclaved at 121°C for 30 min to completely disrupt the tissues and release all electrolytes. The conductivity of the incubation solution with killed tissues (Cmax) was determined after the solution had cooled down to room temperature. The relative EL was calculated using the formula: EL (%) = (Ci/Cmax) × 100.

#### Enzyme activity

For enzyme extracts and assays, The contents were homogenized with 4 ml phosphate buffer (50 mM, pH 7.8) which contained 0.7% NaH_2_PO_4_·2H_2_O and 1.64% Na_2_HPO_4_·12 H_2_O. Enzyme extractions were performed at 4°C. The homogenate was centrifuged at 15,000 × g for 30 min and supernatant was collected.

Soluble protein content was measured according to the method described by Bradford (1976). Briefly, 3 ml of Bradford solution containing 0.01% Commassie Blue G250 (w/v), 4.7% ethanol (v/v), and 8.5% phosphoric acid (v/v) were mixed with 30 μl of supernatant. The absorption of the mixture was determined at 595 nm using a spectrophotometer (UV-2600, UNICO Instruments Co., Ltd., Shanghai, China). Soluble protein content was calculated by the formula obtained by bovine serum albumin standard curve.

The SOD activity was measured as described previously (Giannopolitis and Ries, 1977). The assays were performed at 25°C and the 3 mL reaction mixture consisted of 50 mM phosphate buffer solution (PBS; pH 7.8), 60 mM Riboflavin, 195 mM Met, 3 mM EDTA, 1.125 mM NBT and 0.1 mL enzyme extract. The tested samples were incubated for 10–30 min under 13000 lux irradiance. The absorbance at 560 nm was recorded. One unit of enzyme activity was determined as the amount of the enzyme to reach an inhibition of 50% NBT reduction rate.

Peroxidase activity was determined based on the guaiacol oxidation using H_2_O_2_ (Chance and Maehly, 1955). The reaction mixture consisted of 100 mM sodium-acetic buffer (pH 5.0), 0.25% (w/v) guaiacol and 0.75% H_2_O_2_. Prior to the absorbance measurement at 460 nm, the reaction mixture was fully dissolved and shaken up. One unit POD activity was defined as the absorbance change of one unit per minute.

The CAT activity was determined by measuring the decrease in absorbance at 240 nm for 1 min along with the decomposition of H_2_O_2_ (Chance and Maehly, 1955). The reaction mixture consisted of 50 mM phosphate buffer (pH 7.4) and 45 mM H_2_O_2_ and 0.1 mL enzyme extract. One unit CAT activity was defined as the absorbance change of 0.01 units per minute.

### Determination of H_2_O_2_ content

Quantification of H_2_O_2_ content was determined using the method of Jena and Choudhuri (1981). One millilitre of supernatant was blended in 1 ml of 0.1% titanium sulfate containing 20% H_2_SO_4_(v/v) and then centrifuged for 15 min at 6000 × g. The A_410_ was immediately determined and compared with a standard curve obtained from known concentrations of H_2_O_2_ and calculated using the extinction coefficient of 0.28 μmol^−1^ cm^−1^.

### Quantitative RT-PCR analysis

For gene expression determination, the samples were first stored at −20 °C prepared to the RNA isolation and gene expression analysis. Total RNA was isolated from about 0.1 g crushed leaves using Trizol-reagent (Invitrogen, Carlsbad, CA) according to instructions.

The first strand cDNA was synthesized from 2 μg of total RNA with oligo (dT)_12−18_ primer using cDNA synthesis kit (Fermentas, Canada) according to the manufacturer′s instructions. The gene-specific primers used for real-time quantitative RT-PCR are listed in Table S1. The housekeeping gene, eEF1A(s), was used as control. SYBR Green I (Sigma–Aldrich, US) was used to produce a fluorogenic intercalating dye on a Chromo4 Real-Time Detection System (MJ Research, Cambridge, MA). The quantitative RT-PCR progress for the amplifications was 94°C, 3 min, followed by 38 cycles of 95°C for 10 s, 20 s annealing for different primer at 50–55°C, and 72°C for 20 s, with a final elongation at 72°C for 5 min. 38 cycles, followed by extension at 72°C for 5 min. The PCR products were further operated in a 1.2% agarose gel in 1 × TAE and stained with EtBr, and the band intensity was quantified using imaging software (Tanon 2500, Tanon Science and Technology Co., Ltd., Shanghai, China).

### Statistical analysis

In the experiments, all results were expressed as mean ± *SE* (standard error) of four replicates. The data were subjected to analysis of variance (ANOVA) using the SPSS statistical software package (Ver.16.0, SPSS Inc., Chicago, IL, USA) and DPS v7.05. The graphs were produced using Origin 8.0 (Origin Lab Inc., Hampton, USA) and Adobe Photoshop (Adobe Inc., San Jose, USA). For RT-qPCR, the DDCq method was used to determine the changes of target gene expression based on normalization with the reference gene. Different letters in tables and histograms indicate significant differences between treatments (*P* < 0.05) based on LSD test.

## Results

### Leaf relative water content

When plants were exposed to drought, heat and combination of the two stresses, the RWC in PI 234881 was decreased compared to the control (Table 2). The RWC was reduced by 35.99, 6.47, and 16.1% under drought, heat and a combination of the two stresses, respectively. On the other hand, PI 578718 experienced RWC reduction by 4.4% under drought stress. Under a combination of the two stresses, there was a 7.5% significant RWC decrease in heat tolerant PI 578718, which was greater than in heat sensitive PI 234881.

**Table 2:** **Effects of stress treatments on RWC and Chl content of heat sensitive PI 234881 and heat tolerant PI 578718 under control (C), drought (D), heat (H), combined stress (DH)**.

	**C**	**D**	**H**	**DH**
**—— PI 234881——**				
RWC(%)	98.42±1.86a	63±5.63c	92.05±2.03ab	82.57±1.91b
Chl (mg g ^−1^FW)	35.78±0.282a	23.87±3.11b	31.4±1.32ab	23.79±3.67b
**—— PI 578718——**				
RWC(%)	99.39±1.5a	95.02±0.81bc	96.3±1.19ab	91.94±1.81c
Chl mg g ^−1^FW)	42.33±0.328a	33.67±4.04b	31.77±1.02b	29.5±2.25b

*RWC was measured at the end of stress treatments (14th day) using fully expanded 3rd leaves. Different letters indicate significant differences between treatments (P < 0.05) based on LSD test*.

### Chlorophyll content

Under drought, heat and a combination of the two stresses, the leaf Chl content in PI 234881 decreased compared to the control (Table 2). This decrease was 33.3% in drought, 12.24% in heat and 33.52% in their combination. Notably, the Chl content was significantly affected in PI 578718 by all stress groups. However, the combination stress caused a more severe reduction by 30.31% in Chl content than either stress alone.

### Lipid peroxidation

**Figure 1:**
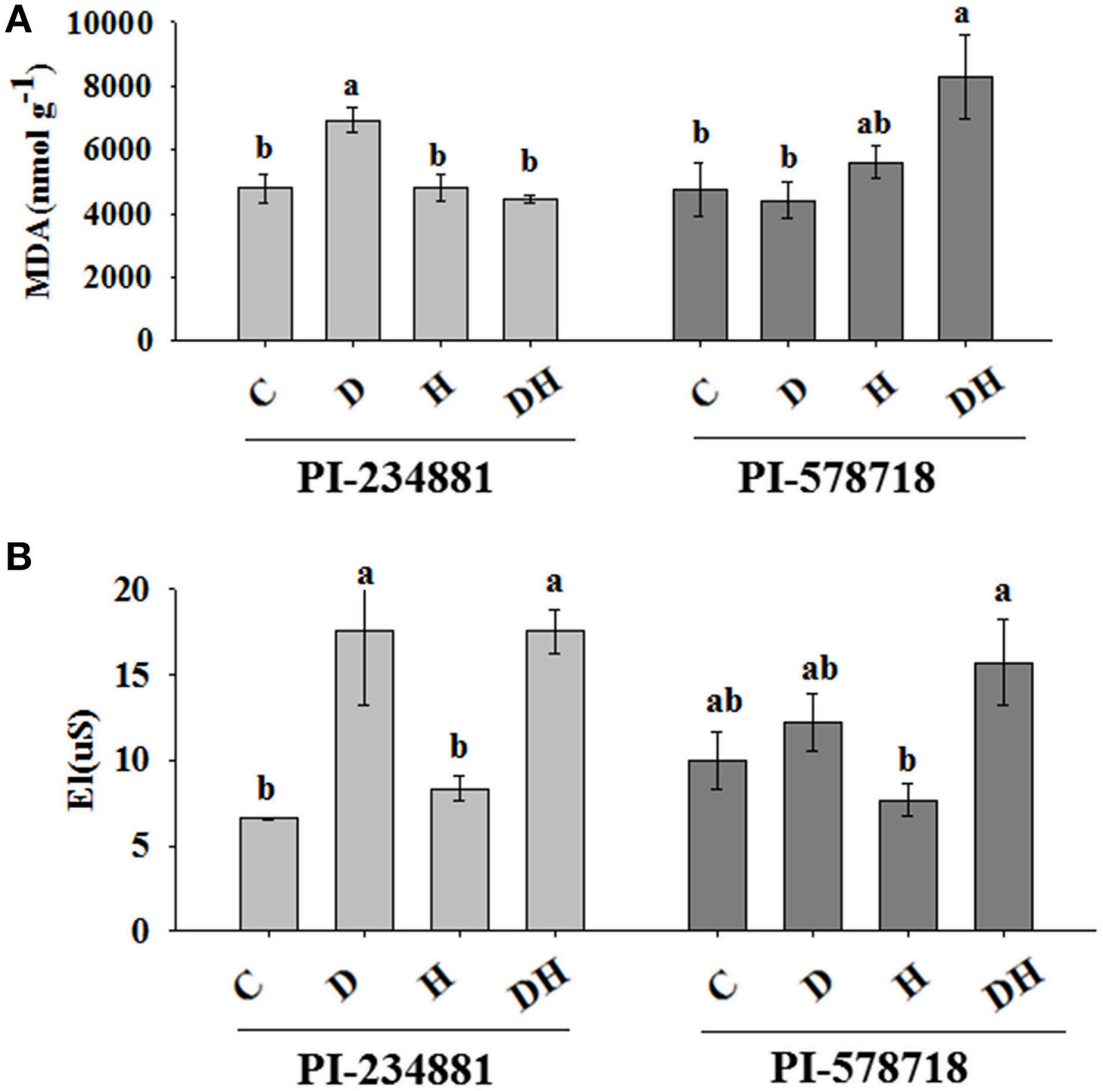
**The effects of stress treatments on MDA content (A) and El level (B) in 7th leaves of PI 234881 and PI 578718 under control (C), drought (D), heat (H), combined stress (DH)**. Different letters indicate significant differences between treatments (*P* < 0.05) based on LSD test.

In the heat sensitive genotype PI 234881, the effect of the combined stress was similar to that of heat stress, while drought stress enhanced the MDA content by 44.91% (Figure 1A). However, in PI 578718 lipid peroxidation was more pronounced under the combined stresses than either stress alone. The combined stresses enhanced the MDA content of PI 578718 by 74.1%.

### Electrolyte leakage

Drought and the combination stresses caused a significant increase in EL in the leaves of the when compared to the control plants (Figure 1B). Under drought and combination stresses conditions, the increase in EL was 22.26 and 57.4% greater in PI 578718, respectively.

### Antioxidant enzyme activities

**Figure 2:**
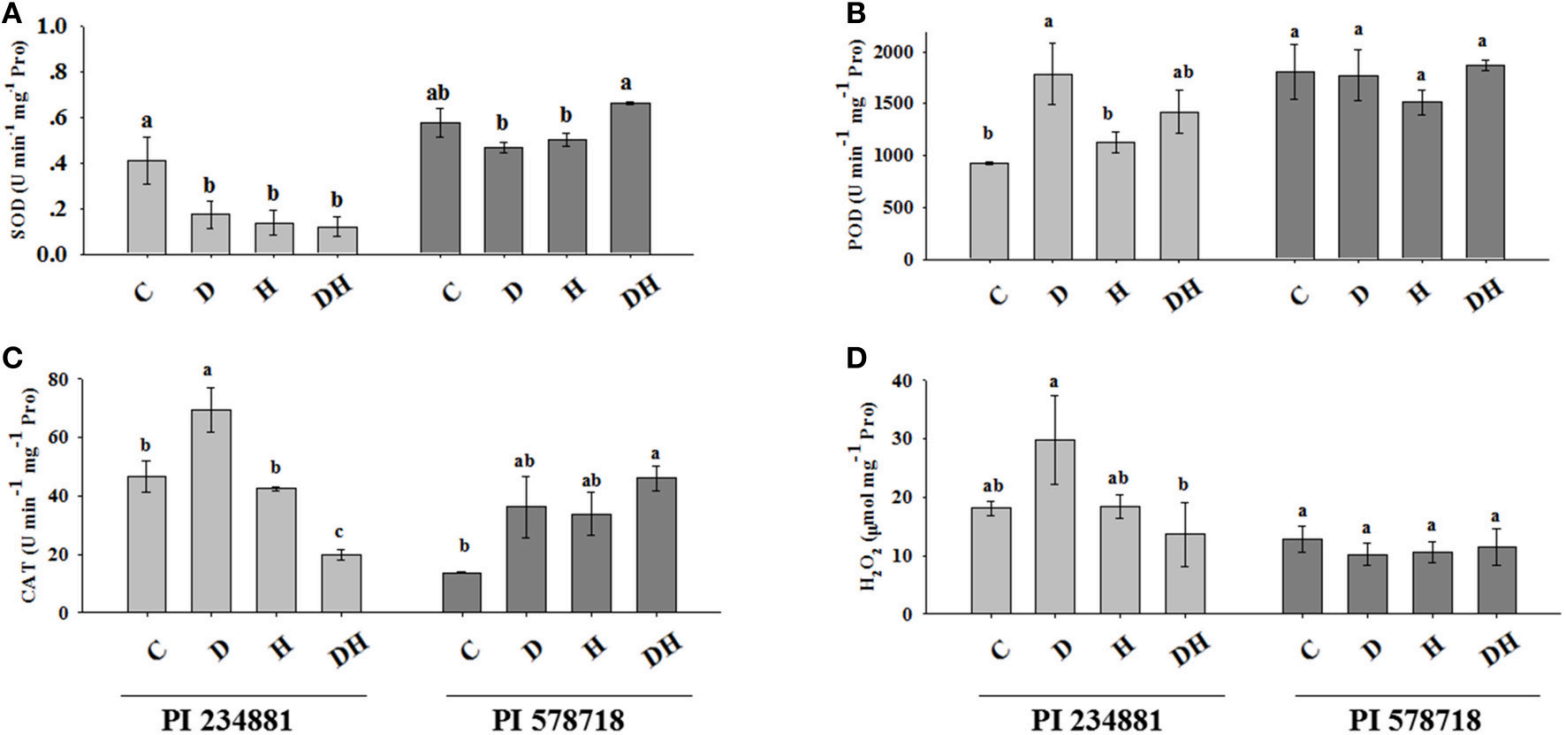
**The effects of stress treatments on SOD (A), POD (B), CAT (C) activities and H_**2**_O_**2**_ (D) content in 7th leaves of PI 234881 and PI 578718 under control (C), drought (D), heat (H), combined stress (DH)**. Different letters indicate significant differences between treatments (*P* < 0.05) based on LSD test.

Generally, the tall fescue under drought, heat and the combination of the two stresses displayed different patterns of the SOD, CAT, and POD activities, when compared to the controls (Figures 2A–C).

The SOD activity in PI 234881 was decreased by 58, 66.4, and 70.95% under drought, heat, and the combination of the two stresses, respectively (Figure 2A). In the heat-tolerant genotype PI 578718, no significant difference in SOD activity was observed between the stress and control regime. However, the SOD activity increased by 15.37% under combined stress as compared with the control, but decreased by 18.92 and 12.35% under drought and heat stress, respectively. The constitutive level of POD in PI 578718 (heat tolerant) was higher than in PI 234881 under controlled conditions (Figure 2B). The POD activity in PI 234881 was increased by 21.33 and 53.12% under heat and the combined stress respectively. On one hand, drought stress in PI 234881 dramatically enhanced the POD activity by 92.61%. There was no notable changes between stress and control group in the tolerant genotype PI 578718. However, the POD activity under the combined stress was enhanced by 3.28% as compared to the control groups. On the other hand, the highest POD activity for the all stress conditions was observed in PI 578718. Drought stress enhanced CAT activity by 48.49% in the PI 234881 while heat stress did not cause any significant change as compared to the control group (Figure 2C). Unexpectedly, combination of stresses decreased CAT activity by 57.45% in PI 234881. In PI 578718, drought and heat stress increased CAT activity by 2.65-folds and 2.47-folds similarly, combined stress significantly enhanced the CAT activity by 3.36-folds as compared to the control group in PI 578718.

### H_2_O_2_ level

Similar to the CAT activity, the H_2_O_2_ content in control regime was higher in PI 234881 compared with PI 578718 (Figure 2D). The H_2_O_2_ content was enhanced in PI 234881 by 64.67 and 2.17% under drought and heat stress, respectively. Conversely, the combined stress decreased the H_2_O_2_ content of PI 234881 by 24.7%. The stress groups had no effects on H_2_O_2_ content in the tolerant genotype PI 578718.

### OJIP fluorescence transient and parameter analysis

**Figure 3:**
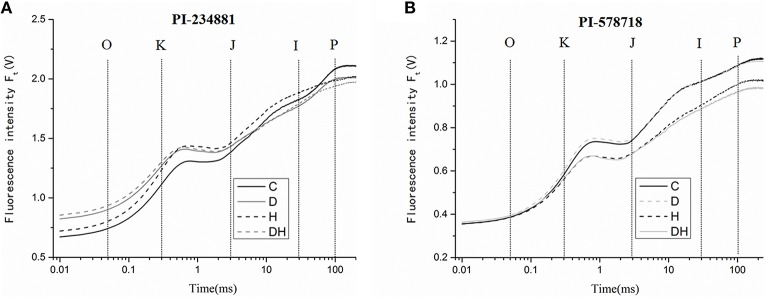
**The OJIP fluorescence transients in tall fescue leaves under control (C), drought (D), heat (H), combined stress (DH) in PI 234881 (A) and PI 578718 (B) accessions**. Tall fescue leaves were vacuum-infiltrated for 15 min in the dark.

Drought, heat, and combination stress treatment significantly affected the OJIP fluorescence transient of tall fescue leaves (Figures 3A,B). The OJIP transient of tall fescue leaves in PI 234881 declined dramatically after dark adaption under drought and heat stresses. The Fm reached its minimum after 7 days drought and heat stress treatment, but the value of F_0_ was significantly enhanced compared to other treatments. In the PI 234881 the relative variable fluorescence value receded by 4.76% under heat stress, but there was no significant difference between drought stress and combination stress (*P* > 0.05). However, in PI 578718 the relative variable fluorescence value was enhanced by 12.82 and 13.68% under heat and the combination stresses, respectively. Comparing the OJIP transient curves of the two tall fescue accessions we uncovered some unique properties. More notably, the overall trend of fluorescence value in PI 234881 was higher than PI 578718. The values from F_0_ to F_k_ are approximated to control treatment in PI 578718.

The value of fluorescence parameters listed in Table 1 was analyzed by the JIP-test to determine the structural and functional parameters quantifying the photosynthetic behavior of the tall fescue leaves. There were significant differences in the functional parameters between control and treatment regimes. In PI 578718, treatment with combination stress in the leaves significantly enhanced the ABS/RC, TR_0_/RC, RE_0_/RC, φ_R0_ ≡ RE_0_/ABS values as compared with the control group (at *P* < 0.05). The group treated with drought and heat also significantly reduced the TR0/ABS, ET_0_/ABS, and PI_ABS_ values at significant level of *P* < 0.05. There were no differences in ET_0_/RC, ET_0_/TR_0_ of tall fescue leaves between control and stress groups. The treatment with drought stress significantly declined the PI_total_ value, which is a crucial index describing the overall activity of PSII. In PI 234881, the stress treatments significantly reduced the ET_0_/RC, φ_P0_ ≡ TR_0_/ABS, ψ_E0_ ≡ ET_0_/TR_0_, φ_E0_ ≡ ET_0_/ABS, φ_R0_ ≡ RE_0_/ABS, and RC/ABS values. More remarkably, the value of PI_ABS_ decreased by 62.03% under drought and heat stress compared to the control group.

### Related gene expression about photosynthetic system

**Figure 4:**
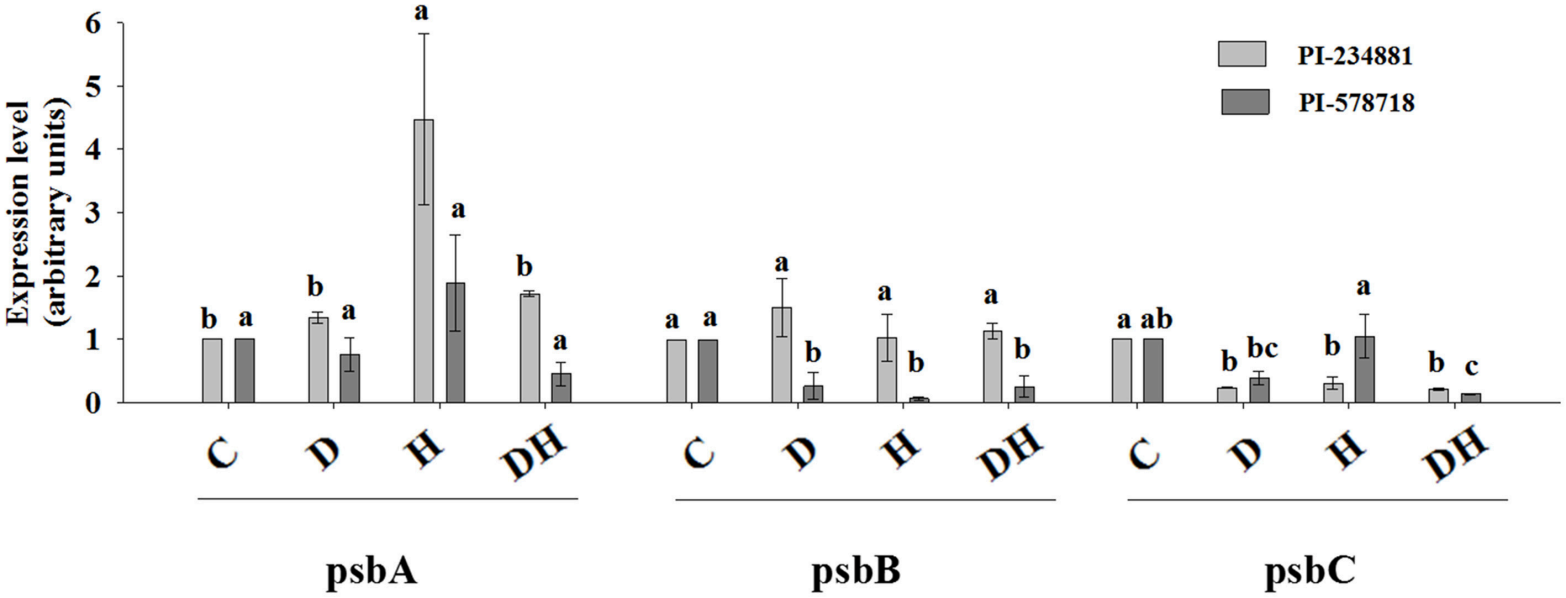
**Effect of drought (D), heat (H), combined stress (DH) on the gene transcription of tall fescue leaves**. Tall fescue leaves were under water (Control), drought (D), heat (H), combined stress (DH) for 7th in the same incubator. Then tall fescue leaves were stored in liquid nitrogen for RT-PCR. Data are given as mean ± *SD* of three independent experiments. Different letters indicate statistical difference significance at *P* < 0.05 among the treatments by Duncan's multiple range tests.

Heat stress significantly enhanced the gene transcription level of *PsbA* (protein subunits of PSII core reaction center complex) subunits relative to the control group in PI 234881 (Figure 4). The correlation among gene expression and chlorophyll a fluorescence transient was found in leaves of tall fescue. Similarly, drought and combined stresses increased the gene transcription level of *PsbA*. However there were no difference in gene transcription level of *PsbA* in PI 578718. The transcription levels under heat stress were higher than those under drought and combined stresses, respectively. Markedly, in the gene transcription level of *PsbB*, stress groups in PI 234881 caused no significant differences compared to the control regime. The gene transcription level of *PsbB* in PI 578718 was more pronouncedly lower under drought, heat and combined stresses than the control and *PsbC* in PI 234881. Notably, in the tolerant genotype PI 578718 the combined stress caused a more severe reduction in the gene transcription level of *PsbC* than either stress alone. The PI 578718 showed less reduction in the gene transcription level of *PsbC* under drought stress. Generally, the gene transcription level was higher under stress conditions in PI 234881 than in PI 578718.

### Associations between physiological index and OJIP kinetic parameters

There was a significant correlation among the physiological index and OJIP kinetic parameter (Table 3). A significant positive correlation was found between Chl and φ_P0_ and PI_ABS_, whereas El was negatively correlated with other trait variables. Particularly, CAT showed a positive correlation with PI_ABS_ in PI 234881. A significant negative correlation was found between SOD and γ_RC_ whereas POD was positively correlated with γ_RC_ in PI 578718.

**Table 3:** **Correlations among Chl, El, SOD, POD, CAT, φ_**P0**_, γ_**RC**_, ψ_**E0**_, PI_**ABS**_, RC/ABS in PI 234881 and PI 578718 tall fescue accessions under drought, heat, and the combined stress**.

	**Chl**	**El**	**SOD**	**POD**	**CAT**	**φ_P0_**	**ψ_*E*0_**	**γ_RC_**	**RC/ABS**	**PI_ABS_**
**—— PI 234881——**										
Chl	1									
El	‒0.624^**^	1								
SOD	0.480	‒0.575^*^	1							
POD	‒0.217	0.451	‒0.370	1						
CAT	0.441	‒0.183	0.022	0.283	1					
φ_P0_	0.704^**^	‒0.581^*^	0.384	‒0.500^*^	0.347	1				
ψ_*E*0_	0.400	‒0.113	0.226	‒0.271	0.400	0.423	1			
γ_RC_	0.595^*^	‒0.457	0.147	‒0.397	0.419	0.948^**^	0.359	1		
RC/ABS	0.251	0.028	0.042	‒0.159	0.459	0.254	0.957^**^	0.265	1	
PI_ABS_	0.638^**^	‒0.460	0.346	‒0.456	0.519^*^	0.846^**^	0.789^**^	0.816^**^	0.704^**^	1
**— PI 578718—**										
Chl	1									
El	‒0.568^*^	1								
SOD	0.062	0.100	1							
POD	‒0.217	0.321	‒0.107	1						
CAT	0.441	‒0.424	0.028	0.283	1					
φ_P0_	0.464	‒0.611^*^	‒0.262	0.239	0.400	1				
ψ_E0_	0.351	0.139	0.350	‒0.215	‒0.011	0.218	1			
γ_RC_	0.037	‒0.163	‒0.492^*^	0.537^*^	0.428	0.801^**^	0.201	1		
RC/ABS	0.132	0.346	0.242	‒0.154	‒0.059	‒0.018	0.903^**^	0.226	1	
PI_ABS_	0.536^*^	‒0.362	‒0.082	0.160	0.343	0.877^**^	0.623^**^	0.770^**^	0.430	1

* *Indicates statistical difference significance at P < 0.05 among the treatments by Duncan's multiple range tests*.

** *Indicates statistical difference significance at P < 0.01 among the treatments by Duncan92s multiple range tests*.

## Discussion

In this study, we characterized the particular antioxidant metabolism and PSII photochemistry response pattern to drought, heat and a combination stress in two tall fescue accessions with different high-temperature tolerance. We found that high-temperature sensitive PI 234881 exhibited higher EL, MDA, and H_2_O_2_ content, and the lower leaf Chl content compared to high-temperature tolerant PI 578718 under drought or heat stress or a combination stress. These results indicated that different abiotic stress conditions generated the fundamental differences between the acclimation responses to stress conditions in different plant sepecies, which provides adaptive flexibility to enhance survival potential.

Drought, heat, or a combination stress can generate the impact of varying degrees on RWC, Chl, MDA, and EL level in cool-season turfgrass (Huang et al., 1998). Shi et al. (2012) reported that different drought tolerant bermudagrass accessions had high natural variation of physiological traits and drought sensitive bermudagrass (Yukon) showed relative lower LWC, higher EL and more accumulation MDA than drought tolerant bermudagrass (Tifgreen). Here, RWC of the sensitive variety PI 234881 was more affected by drought alone, rather than by the combination, we hypothesis that the sensitive variety first stand a drought stress, and after a period of acclimation it then adatpted the enviroment. In addition, drought, heat, or the combined stresses generated significant declines in leaf Chl in both tall fescue genotypes. Heat-tolerant genotype PI 578718 maintained comparatively higher Chl content, and fewer decline compared to PI 234881. A sharp fall of Chl content would decrease the chloroplast biochemistry or Chl fluorescence. Drought-induced stress weakened photosynthetic electron transfer and Chl contents as reported previously (Zuily-Fodil et al., 1990; Moran et al., 1994). The loss of Chl contents during stress measurements could also be related to photo-oxidation resulting from oxidative stress.

El and MDA were valuable indicators of the degree of cellular damages caused by environment stress (Hernández and Almansa, 2002). In the present study, a pronounced increase in EL for PI 234881 was observed when subjected drought and the combined stress. This suggests that an increase in membrane permeability or loss of membrane stability might lead to increased solute leakage. However, the EL in PI 578718 was 95.5% lower than in PI 234881, which suggested that the membrane permeability of heat-tolerant PI 578718 was less affected by drought stress, and had more enhanced protective mechanisms. These results are consistent with Bajji et al. (2001), who reported the mechanisms of higher membrane stability stress in durum wheat under water stress. In addition, consistent with Sekmen et al. (2014), our study indicated that there was less lipid peroxidation in PI 23488l genotype under combined stress. However, in PI 578718 there was more lipid peroxidation under combined stress than drought stress alone. This observation is consistent with previous studies (Jiang and Huang, 2000). The increase observed in leaf MDA contents of both genotypes after drought, heat and the combined stresses were in line with results from others (Price and Hendry, 1991; Zhang et al., 1995). In both tall fescue genotypes, the MDA content was the least affected parameter in the course of the entire experimental stress treatments. This indicated that the adaptabilities to drought and heat could be linked with minimum lipid peroxidation and high antioxidant enzymes antioxidant activities, particularly CAT.

Drought, heat, or the combined stress can also have an effect on varying degrees on ROS in cool-season turfgrass (Huang et al., 1998). Induction of antioxidant enzyme defense activities is highly correlated with increased environmental stress (Ashraf, 2009). Cellular SOD constitutes the first line of defense against ROS (Alscher et al., 2002), and it can catalyze the dismutation of superoxide radical (${\text{O}}_{2}^{-∙}$) to oxygen and (H_2_O_2_; Jaleel et al., 2009). The higher SOD activity in PI 578718 (heat tolerant) under the drought and heat stress could be a consequence of excess superoxide radical (${\text{O}}_{2}^{-∙}$) generation. Superoxide radical has been regarded as a signal for antioxidant enzyme induction, and consequently, it might have resulted in greater induction of SOD (Almeselmani et al., 2006). This result indicated that the genotype PI 578718 had a high capacity to catalyze the dismutation of ${\text{O}}_{2}^{-∙}$ to H_2_O_2_ and O_2_ under drought and heat stress. Although, there is precise report regarding the scavengers of oxygen or the hydroxyl radical (^.^OH), SOD plays a functional role in their elimination by chemical reaction (Alvarez, 2000).

POD and CAT are among the major enzymes that scavenge H_2_O_2_ which is produced through the dismutation of ${\text{O}}_{2}^{-}$ in peroxisomes and chloroplasts. Drought stress increased the POX activity in soybean plants (Zhang et al., 2006). In the present study, the POD activity in heat-tolerant genotype PI 578718 under drought and heat stress was enhanced by a little 3.28% as compared to the control groups. Generally, PI 578718 showed considerably greater POD activity than PI 234881, which indicated that the former genotype has better scavenging ability and higher tolerance to drought and heat stress than the latter. These observations are consistent with the results of Almeselmani et al., (2006). In the present study, although CAT activity of heat-sensitive PI 234881 was enhanced under drought stress, its activity declined under the combined stress. This may be attributed to the increased H_2_O_2_ accumulation and lipid peroxidation which may have excited CAT and POD activities. These findings are almost the same consistent with what Sekmen et al. (2014) observed. As we have documented, the H_2_O_2_ content in drought-tolerant PI 234881 was higher than that in PI 578718. We hereby hypothesize that this is relevant because of CAT activity in PI 578718. The cooperation of POD and CAT in ROS scavenging is more complex, and might involve other peroxidases. The comparison of the heat-sensitive PI 234881 and heat-tolerant PI 578718 response pattern for SOD, CAT, and POD activities to drought and heat stress indicated significant difference at different abiotic stress. Relatively heat-tolerant PI 578718 showed increased or decreased enzyme activities compared sensitive genotype PI 234881, which maybe is correlated with the temporal regulation of the constitutive isoenzymes as well as antioxidant genes.

Drought, heat, or the combined stress can exert an effect on varying degrees on PSII in chloroplast (Čajánek et al., 1998; Crafts-Brandner and Salvucci, 2002; Musil et al., 2009). The rise in chlorophyll fluorescence induction reveals a characteristic O-J-I-P polyphasic transient (Strasser et al., 2004). The performance index PI_total_ (performance index for energy conservation from exciton to the reduction of PSI end acceptors) is the most sensitive parameter of the JIP-test in evaluating plant photochemical activities under stressful condition. It incorporates several parameters that are evaluated from the fluorescence transient OJIP (Clark et al., 2000; Yusuf et al., 2010). From the JIP test, the maximum quantum yield for primary photochemistry (φ_P0_ ≡ TR_0_/ABS), the quantum yield of electron transport (φ_E0_ ≡ ET_0_/ABS) and the motion of a trapped exciton into the electron transport chain beyond Q_A_ (ψ_0_ ≡ ET_0_/TR_0_) could be estimated. The φ_P0_ and φ_E0_ values were markedly changed under drought and heat stress. However, the ψ_0_ values were slightly altered under stress treatments. Our previous study showed that the behavior of PSII among six heat-tolerant accessions and five heat-sensitive accessions had various thermotolerance capacities in response to high-temperature stress (Chen et al., 2014). In this study, we also found the similar tendency that heat-tolerant PI 578718 obtained 12.82% increase of the relative variable fluorescence value but heat-sensitive PI 234881 receded by 4.76% the relative variable fluorescence value. Furthermore, Hagemeyer (2004) and Perales-Vela et al. (2007) reported that to protect the plant growth from extremely adverse environmental condition, excess excitation energy was transformed into thermal dissipation so as to maintain the energy balance between absorption and utilization. And the PI_total_ value under heat and the combined stress also confirmed this. However, the PI_total_ value under drought stress had a profound change. Therefore, we deduced that this discrepancy maybe on account of technical complexities and errors sample collection method, hence the results should be evaluated with maximum caution (Queval et al., 2008). For example, under heat stress, plants open their stomata to cool their leaves via transpiration. However, if heat stress is combined with drought, plants would not open the stomata and the leaf temperature would be higher (Rizhsky et al., 2002). Under single stress treatment φ_P0_, ψ_0_, φ_E0_, and PI_ABS_ values had no significant difference compared to the control group. Researchers reported that drought did not have a significant impact on the photosynthetic efficiency, and therefore photosynthesis was significantly compromised under heat stress. It was demonstrated that mild drought affected plant growth, but had subtle effects on the photosynthesis rate in *Arabidopsis* (Muller et al., 2011; Skirycz et al., 2011; Verelst et al., 2013).

CP43 (*psbB* encoded protein) and CP47 (*psbC* encoded protein; light-harvesting complex) are the intrinsic transmembrane proteins which are located in the reaction center of PSII.. In the present study, the transcription of CP43 and CP47 under drought, heat and the combined stress in PI 578718 and PI 234881 tall fescue genotypes decreased significantly. This resulted in the weakened RC, and thus confirmed that heat and the combined stress could accelerate photosynthesis. After CP47 was released from the reaction center (RC), light harvesting antenna would be uncoupled out of the RC and the damaged D1 protein would be cleavaged under detrimental environmental heat (Yoshioka et al., 2006). In the present study the expression of D1 protein is higher in PI 234881 than that in PI 578718 when under each stress group. The results indicated that the increase in *psbA* expression is benefitial to PSII RC by resisting drought and heat stress and improving the PSII recovery. As Takahashi et al. (2004) reported that the presence of highly reactive singlet oxygen in OEC is detrimental to the D1 protein, and heat stress enhances the susceptibility of this photosynthetic organs. Therefore, the novel expression of psbA is a supplement for the damaged D1 protein and is fairly vital for PSII. Of course, the gene expression of *psbA, psbB*, and *psbC* is not enough. Wang et al. (2015) have researched that Hsfs and their target genes in *F. arundinacea* and *L. perenne* also provied a foundation for future gene function studies to improve stress tolerance in grasses and other crops. Yang et al. (2015) also have reported that *TIP41* combined with *TUB* and *ACT* was stably expressed in heat-stressed leaves and these suitable reference genes in tall fescue would allow for more accurate identification of stress-tolerance genes in this stress-tolerant species.

In the present study, differential adaptations in different plant species under different abiotic tolerance can have differential natural variation of physiological traits. Relatively heat-tolerant PI 578718 experienced greater increase or less decrease of enzyme activities compared to the heat-sensitive PI 234881. This may be correlated with the lower H_2_O_2_ content as well as antioxidant genes. Generally, drought, heat and the combined stresses triggered oxidative injury on diffirent levels in both tall fescue genotypes, as showed by the reduction in antioxidant enzymes (e.g., SOD activity in 578718 and SOD and CAT activity in PI 234881), and increase in lipid peroxidation. Drought did not have significant impact on the photosynthetic efficiency i.e., photosynthesis was significantly compromised under heat and the combined stress. These results broadened our comprehension on various enzymatic antioxidant metabolism and PSII acclimation to drought, high-temperature and a combination of both stresses in cool-season turfgrass species. And the specific molecular mechanism underlying improved growth traits and stress tolerance is our further study.

## Author contributions

AB carried out the experiments and wrote the manuscript. JF and ZH analyzed the data. GW assisted with doing the experiments. JF and TH. conceived and designed the experiments. TH and EA helped to draft the manuscript and revise the manuscript. All authors read and approved the final manuscript.

### Conflict of interest statement

The authors declare that the research was conducted in the absence of any commercial or financial relationships that could be construed as a potential conflict of interest.

## Abbreviations

CAT: Catalase
Chl: Chlorophyll
DW: Dry weight
EL: Electrolyte leakage
FW: Fresh weight
MDA: Malondialdehyde
OEC: Oxygen-evolving complex
POD: Peroxidase
PSI: Photosystem I
PSII: Photosystem II
PQ: Plastoquinone
ROS: Reactive oxygen species
RWC: Relative water content
SOD: Superoxide dismutase.
