# Supplementary material for: Differential Acclimation of Enzymatic Antioxidant Metabolism and Photosystem II Photochemistry in Tall Fescue under Drought and Heat and the Combined Stresses
Source: Front Plant Sci. 2016 Apr 14;7:453. doi: 10.3389/fpls.2016.00453 (PMC4830848; doi:10.3389/fpls.2016.00453)
Supplement: Supplementary file 1 [file Table1.DOC]

**TABLE S1 Primer sequences and information used for reversed transcription real-time PCR (RT-PCR) analyses.**

| Gene | Encoded polypeptide |  | Primers sequences (5’–3’) | Size (bp) | Gene ID |
| --- | --- | --- | --- | --- | --- |
| *psb*A | D1 protein | F | GTATTTATTATCGCCTTCATCG | 284 | 7095419 |
|  |  | R | AGGACGCATACCCAAACG |  |  |
| *psbB* | CP43 | F | TAATACGGCTTATCCGAGTGAGTTT | 288 | 7095484 |
|  |  | R | TCTTGCCAAGGTTGTATGTCTTTT |  |  |
| *psbC* | CP47 | F | TAGGCGTAACGGTGGA | 254 | 7095420 |
|  |  | R | AACATCTCGGAACAAGG |  |  |

F and R represent forward and reverse, respectively.
